# Histochemical Characterization of the Vestibular Y-Group in Monkey

**DOI:** 10.1007/s12311-020-01200-z

**Published:** 2020-10-20

**Authors:** Christina Zeeh, Ümit S. Mayadali, Anja K.E. Horn

**Affiliations:** 1 Institute of Anatomy and Cell Biology, Dept. I, Ludwig-Maximilians University, Pettenkoferstrasse 11, Munich, 80366 Germany; 2 Graduate School of Systemic Neurosciences, Großhaderner Str. 2, Planegg, 82152 Germany

**Keywords:** Vestibulo-ocular reflex, Floccular-target neurons, Smooth pursuit, Voltage-gated potassium channels, Calretinin, Glutamate decarboxylase

## Abstract

The Y-group plays an important role in the generation of upward smooth pursuit eye movements and contributes to the adaptive properties of the vertical vestibulo-ocular reflex. Malfunction of this circuitry may cause eye movement disorders, such as downbeat nystagmus. To characterize the neuron populations in the Y-group, we performed immunostainings for cellular proteins related to firing characteristics and transmitters (calretinin, GABA-related proteins and ion channels) in brainstem sections of macaque monkeys that had received tracer injections into the oculomotor nucleus. Two histochemically different populations of premotor neurons were identified: The calretinin-positive population represents the excitatory projection to contralateral upgaze motoneurons, whereas the GABAergic population represents the inhibitory projection to ipsilateral downgaze motoneurons. Both populations receive a strong supply by GABAergic nerve endings most likely originating from floccular Purkinje cells. All premotor neurons express nonphosphorylated neurofilaments and are ensheathed by strong perineuronal nets. In addition, they contain the voltage-gated potassium channels Kv1.1 and Kv3.1b which suggests biophysical similarities to high-activity premotor neurons of vestibular and oculomotor systems. The premotor neurons of Y-group form a homogenous population with histochemical characteristics compatible with fast-firing projection neurons that can also undergo plasticity and contribute to motor learning as found for the adaptation of the vestibulo-ocular reflex in response to visual-vestibular mismatch stimulation. The histochemical characterization of premotor neurons in the Y-group allows the identification of the homologue cell groups in human, including their transmitter inputs and will serve as basis for correlated anatomical-neuropathological studies of clinical cases with downbeat nystagmus.

## Introduction

The Y-group plays an important role in the generation of smooth pursuit (upward) eye movements [1], but is also involved in the adaptive properties of the vertical vestibulo-ocular reflex (VOR) for example in response to mismatch of visual and vestibular input by wearing optical devices, or in VOR suppression during combined eye-head tracking [2, 3]. Recording studies in monkey revealed a rather uniform neuron population in the Y-group that modulated in phase with eye velocity during visual following, modulated in phase with head velocity during VOR suppression, but did not modulate during the VOR in darkness [4]. Accordingly, the Y-group is part of a circuitry that uses vestibular and visual signals mediated by the cerebellar flocculus to control eye movements. Malfunction of this circuitry may cause eye movement disorders, such as downbeat nystagmus seen after floccular lesions [5].

The Y-group is a well-defined nucleus in the cerebellar white matter at the level of the cerebellomedullary junction described in several mammalian species [6]. Cytoarchitecturally, it consists of a dorsal larger-celled Y-group (Yd) corresponding to the infracerebellar nucleus [7] and a small-celled ventral Y-group (Yv) [8, 9]. Only the Yv receives direct input from saccular afferents [10–12], has commissural connections to the contralateral vestibular nuclei and Yv [13–16] and projects to the flocculus [17, 18]. The Yd receives a disynaptic excitatory input from the ipsilateral anterior and posterior semicircular canals via neurons in the anterior-lateral corner of the superior vestibular nucleus (SVN) and caudal medial vestibular nucleus (MVN) carrying head velocity-only signals [19]. Neurons in the Yd further receive a strong inhibitory input from Purkinje cells of the flocculus (vertical optokinetic and visual-related zones F1 and F3) and ventral paraflocculus; they are therefore termed floccular-target neurons (FTN) [17, 20–22]. The Yd contains a large population of premotor neurons, which includes excitatory neurons targeting motoneurons for upgaze in the contralateral oculomotor nucleus (nIII), as well as neurons with ipsilateral projections to motoneurons in nIII and trochlear nucleus (nIV) involved in downgaze, which presumably have an inhibitory function [7, 14, 23–25]. Our previous studies indicated that the excitatory projections of Yd to nIII are associated with the calcium-binding protein calretinin (CR) [26, 27]. In the present study, we aimed to delineate contralaterally and ipsilaterally projecting neurons by their histochemical properties and by investigating the presence of different cellular proteins, which are related to firing characteristics and transmitters (calretinin, GABA-related proteins and ion channels).

## Material and Methods

The tissue of seven rhesus monkeys (*Macaca mulatta*) from previous studies stored either in a mixture of glycerol and phosphate buffer at − 20 °C or embedded in paraffin was used for staining in the present investigation.

All experimental procedures involving tracer injections had conformed to the state and university regulations on Laboratory Animal Care, including the Principles of Laboratory Animal Care (NIH Publication 85-23, Revised 1985), and were approved by the Animal Care Officers and Institutional Animal Care and Use Committees at Emory University and University of Washington, where all surgical interventions and perfusions were made for previous studies [27–29]. The brains of all cases were fixed by transcardial perfusion with 4% paraformaldehyde (PFA) in 0.1 M phosphate buffer except one case, which was fixed with 2% PFA and 0.5% glutaraldehyde (see Table 1).

**Table 1:** Table 1

Case	Injection	Fixation	Sections	IF	IHC
TC1	1% CTB in right nIII	4%PFA	Frozen	gCTB+mCR+rGAD gCTB+rCR+mGAD gCTB+rCR, gCTB+rGAD	
TC2	5% WGA in left nIII	4% PFA	Frozen	gWGA+mNPNF, gWGA+mACAN, gWGA+rGAD, gWGA+rCR	WGA
PF1		4% PFA	Paraffin		CSPG, NPNF, rCR+mACAN, mCR+rGAD, Kv3.1b, Kv1.1
PF2		4% PFA	Paraffin		rCR+mACAN, mCR+rGAD, Kv3.1b, Kv1.1
PF3		4% PFA	Paraffin		rCR+mACAN, mCR+rGAD, Kv3.1b, Kv1.1
M1		4% PFA	Frozen		sGAD+Nissl
M2		4% PFA	Frozen		rCR+Nissl,
M3		4% PFA	Frozen	rGAD+mCR, mCB+rCR	gChAT
M4		2% PFA, 0,5% GA	Vibratome		rGABA
M5	2,5% WGA-HRP	4% PFA	Frozen		*

An overview of injection, fixation and immunohistochemistry details for each case. *WGA-HRP was visualized by tetramethylbenzidine reaction

The brain sections of two rhesus monkeys (*Macaca mulatta*) from a previous study, who had received a central tracer injection with either 1% cholera toxin subunit B (CTB, Sigma/Biological laboratories, TC1) into the right oculomotor nucleus (nIII) or with 5% wheat-germ agglutinin (WGA; EY Lab, San Mateo, CA; TC2) into the left nIII were used (for details see [27, 28]). Frozen free-floating sections (40 μm) of one additional case was used for double-immunofluorescence staining (M3), and immunoperoxidase staining for different markers was performed in neighbouring thin sections (5 μm) of three paraffin embedded cases (PF1, PF2, PF3). Immunostained sections from previous two cases (M1, M4) treated with a polyclonal sheep glutamate decarboxylase (GAD) and rabbit GABA antiserum were analysed for GABAergic neurons in Yd for validation. A section from a case with a large WGA-HRP injection into nIII (M5) served to illustrate the complete population of premotor neurons in Yd (see Table 1).

### Visualization of the Tracer Combined with Different Markers

In selected sections of the two tracer cases (TC1, TC2) combined immunofluorescence labelling was performed for the simultaneous detection of WGA or CTB and different markers as described previously [27]: Free floating sections were incubated in a cocktail containing either goat antiWGA (1:250, AB_2315611) or goat antiCTb (1:5000, AB_10013220) and one or two of the following antibodies for 48 h at 4 °C: rabbit (1:500, AB_90715) or mouse anti-glutamate decarboxylase (GAD65/67) (1:500, AB_10710523) to reveal GABAergic profiles, mouse anti-nonphosphorylated neurofilaments (NPNF) (1:2500, AB_2715852), mouse anti-aggrecan (ACAN) (1:25, AB_972582) to stain perineuronal nets (PN) and rabbit or mouse anti-calretinin (CR) (1:1000, AB_10000320; 1:2000). After washing, the sections were treated with a mixture of Alexa 488 tagged donkey anti-goat (1:200; AB_2534102) and Cy3-tagged donkey anti-rabbit (1:200, AB_2307443) or Cy3-tagged donkey anti-mouse (1:200, AB_2687868) for 2 h at room temperature. For triple immunofluorescence, either CTB or CR was detected with DyLight™ 405 tagged secondary antibodies (donkey anti-goat IgG, AB_2340426 or donkey anti-rabbit IgG, AB_2340616).

### Immunofluorescence Staining for Different Markers

To validate the findings of tracer-labelled neurons, we investigated, whether CR-positive neurons coexpressing GAD-immunoreactivity are present. For that cryosections of case M2 were incubated in a cocktail with mouse anti-CR (1:2000, AB_10000320) and rabbit antiGAD65/67 (1:500, AB_90715). The antigenic sites were detected by treating sections with a mixture of Alexa 488 tagged donkey anti-mouse (1:200; AB_2341099) and Cy3-tagged donkey anti-rabbit (1:200, AB_2307443) as described previously [26]. Additional sections were incubated in a cocktail with mouse anti-GAD (1:500, AB_10710523), rabbit anti-calbindin (CB) (1:1000, AB_10000340) and goat anti-hyaluronan and proteoglycan link protein 1 (HPLN) (1:100, AB_2116135) revealing PNs to study coexpression of both markers in synaptic boutons attached to neurons in the dentate nucleus and premotor neurons in Yd.

### Immunoperoxidase Staining of Paraffin Sections

Sets of neighbouring 5 μm thick paraffin sections of 3 monkey cases (PF1, PF2, PF3) were stained with immunoperoxidase methods either for the detection of CR and GAD, CR and ACAN, or potassium channels Kv1.1 or Kv3.1b, chondroitin-sulphate proteoglycans (CSPG) or NPNF. After dewaxing and rehydration sections were rinsed in distilled water and reacted in 0.01 M sodium citrate buffer (pH 6.0) at 1160 w in microwave (AEG, Micromat) three times for 3 min. After cooling down to room temperature sections were rinsed in distilled water and transferred to Tris buffered saline (TBS; pH 7.6) for subsequent immunostaining. Single immunoperoxidase detection of the potassium channels was achieved by incubating the sections in either rabbit anti-Kv1.1 (1:750, AB_2040144) or rabbit anti-Kv3.1b (1:6000, AB_2040166) for 48 h at 4 °C. Neighbouring sections were immunostained for either the combined detection of CR and PN or CR and GAD. This was achieved by processing sections with rabbit anti-GAD (rGAD; 1:2000, AB_90715) or mouse anti-ACAN (1:75; AB_972582), with subsequent incubation in biotinylated secondary anti-rabbit IgG (1:200; AB_2336201) or anti-mouse IgG (1:200, AB_2313581) and extravidin peroxidase (EAP) (1:1000; Sigma, St. Louis, MO) visualized with diaminobenzidine-(DAB)-Ni to yield a black reaction product. For the subsequent detection of CR, sections were incubated in either mouse anti-CR (1:2000; AB_10000320) or rabbit antiCR (1:2500, AB_10000321). The antigenic sites were visualized with incubations in biotinylated horse anti-mouse or anti-rabbit (1:200; AB_2313581 or AB_2336201) followed by EAP and a final DAB reaction to yield a brown reaction product. Other dewaxed sections were treated with either mouse anti-chondroitin sulphate proteoglycan (CSPG; 1:500, AB_2219944) or mouse anti-NPNF (1:5000, AB_2715852), followed by an incubation in biotinylated anti-rabbit (1:200; AB_2336201) and EAP with subsequent DAB-Ni reaction. In additional sections cholinergic neurons were detected by immunostaining for choline acetyltransferase (ChAT) as described previously [30]. To confirm the presence of GABAergic neurons in the Y-group, sections that had been immunostained with sheep anti-GAD [31] or mouse anti-[7]GABA [32] from previous studies were evaluated [33, 34].

### Analysis of Stained Sections

The slides were examined and analysed with a Leica microscope DMRB (Bensheim, Germany). Photographs were taken with a digital camera (PixeraPro 600ES; Klughammer, Markt Indersdorf, Germany) mounted on the microscope. The images were captured on a computer with PixeraViewfinder software (Klughammer) and processed with Photoshop 7.0 (Adobe Systems, Mountain View, CA, USA). In each complete image, the sharpness, contrast and brightness were adjusted using the ‘unsharp mask’ and ‘levels adjustment tool’ of Photoshop until the appearance of the labelling seen through the microscope was achieved. The images were arranged and labelled with CorelDraw (version18.0; Corel Corporation, SCR_014235).

Images from selected immunofluorescence preparations were taken with a laser-scanning confocal microscope (Leica SP5, Mannheim, Germany) at × 20 or × 63 magnification. Dual and triple imaging of Alexa 488, Cy3 and Dylight was sequentially recorded at 488 or 543 or 405 nm excitation wavelength, respectively. *Z*-series were collected every 0.5 μm (at × 63) or 1 μm (at × 20) through each section. Image stacks were processed with Fiji/ImageJ software (https://imagej.net/Fiji, SCR_003070). Contrast and brightness of the final composite images were adjusted to reflect the appearance of the labelling, seen through the microscope by using Fiji software.

Immunoperoxidase staining of paraffin sections was imaged using a slide scanner (Mirax MIDI, Zeiss) equipped with a Plan-Apochromat objective (Zeiss, × 20). The digitized images were viewed and photographed with the free software Panoramic Viewer (3DHistech; 1.152.3) and Case Viewer (3DHistech; v2.2). The corresponding detailed views of equally arranged and magnified images of neighbouring sections were analysed on the computer screen. The same neurons were identified by their location with the help of anatomical landmarks, e.g. capillaries.

The cell size profile of the GABAergic and CR-positive neurons within the complete Y-group neuron population was revealed with Fiji/ImageJ software by outlining the somata in Nissl- and immunostained sections at the focus plane of the cell nucleus. The cell sizes were calculated as mean diameter (dmin+dmax/2) and histograms were created with Excel (2016; Microsoft).

## Results

**Fig. 1:**
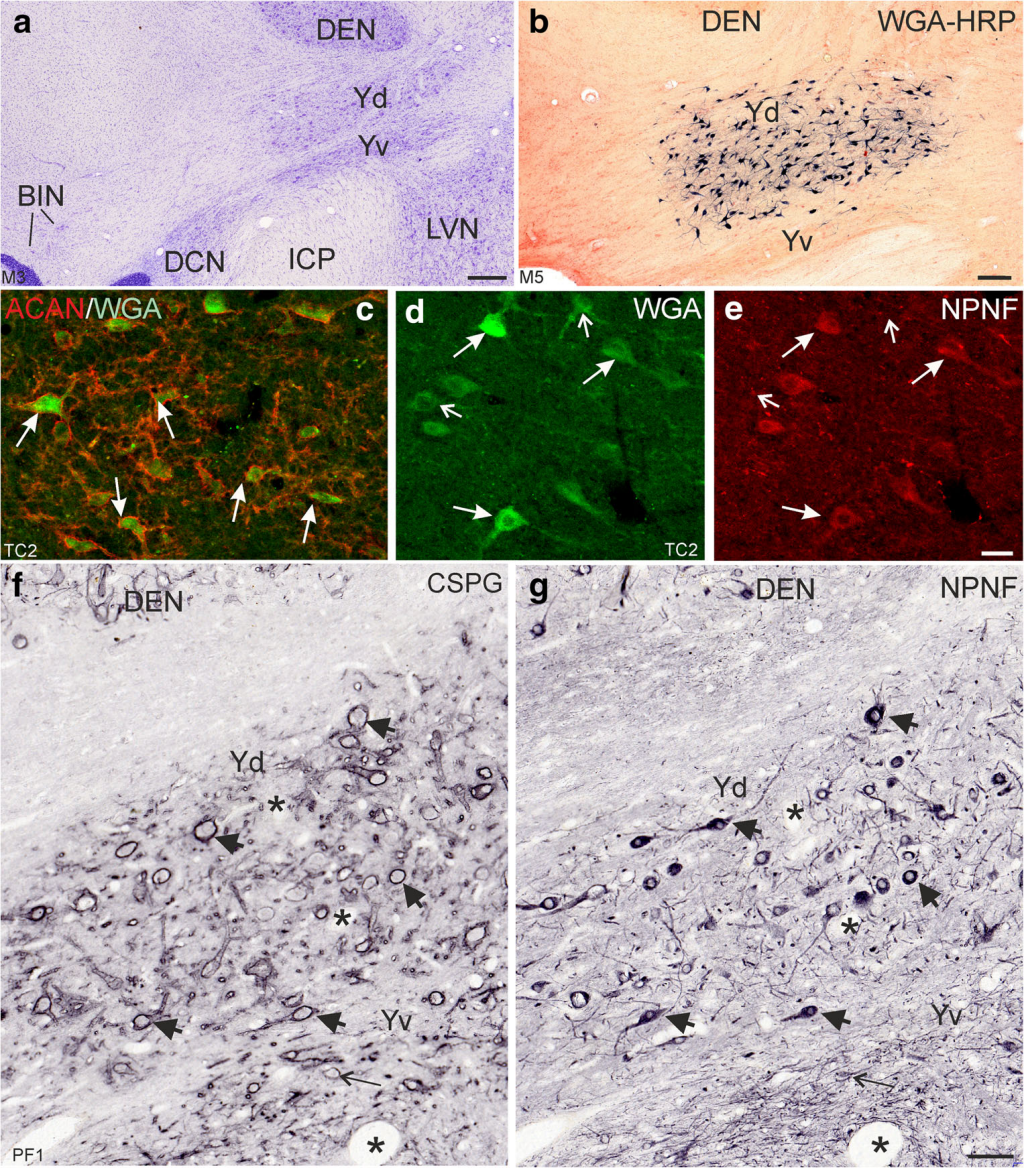
**a** Transverse section through the brainstem of monkey to demonstrate the location of the Y-group with its ventral (Yv) and dorsal (Yd) subdivisions. **b** Overview of transverse section showing retrogradely labelled neurons only in the Yd after WGA-HRP injection into the oculomotor nucleus. **c** Double immunofluorescence staining for WGA (green) and aggrecan (ACAN)-based perineuronal nets (red) demonstrates that all tracer-labelled cells are ensheathed by perineuronal nets (white arrows). **d**, **e** Similarly,  except few (small arrows) tracer-labelled neurons (**d**) express immunoreactivity for nonphosphorylated neurofilaments (NPNF) (**e**, long arrows). **f**, **g** Neighbouring 5 μm thin frontal sections of the Y-group immunostained for chondroitin-sulphate proteoglycans (CSPG) (**f**) or NP-NF (**g**) demonstrates that both markers are coexpressed in medium-sized neurons in the dorsal Y-group (Yd) (**f,g**, large arrows). Smaller neurons in the ventral Y-group (Yv) are ensheathed by perineuronal nets as well , but express only weak NPNF-immunoreactivity (**f, g**, thin arrow). The asterisks label the same blood vessels in both sections as a landmark. BIN, basal interstitial nucleus of the cerebellum; CTB, cholera toxin subunit B; DCN, dorsal cochlear nucleus; DEN, dentate nucleus; LVN, lateral vestibular nucleus; WGA, wheat germ agglutinin; Yd, Y-group dorsal part, Yv, Y-group ventral part; ICP, inferior cerebellar peduncle. Scale bar = 500 μm in **a**, 200 μm in **b**, 50 μm in **e** (applies to **c**–**e**), 100 μm in **g** (applies to **f**–**g**)

In Nissl-stained sections, see Fig. 1a, the two subdivisions of the Y-group are clearly outlined by their cytoarchitecture. The dorsal Y-group (Yd) is composed of loosely packed mainly medium-sized multipolar neurons with mean diameters between 20 and 40 μm, with less small neurons (mean diameter between 10 and 20 μm; see Fig. 4), whereas the ventral Y-group (Yv) consists of tightly packed smaller neurons overlying the inferior cerebellar peduncle (ICP) [35]. At planes of the cerebello-medullary junction the Yd is bordered dorsally by the dentate nucleus (DEN), and lateral by the fibres of the floccular peduncles, which contain the scattered neurons of the basal interstitial nucleus of the cerebellum (BIN) (Fig. 1a) [36].

### Properties of Projection Neurons to Oculomotor Nuclei

**Fig. 2:**
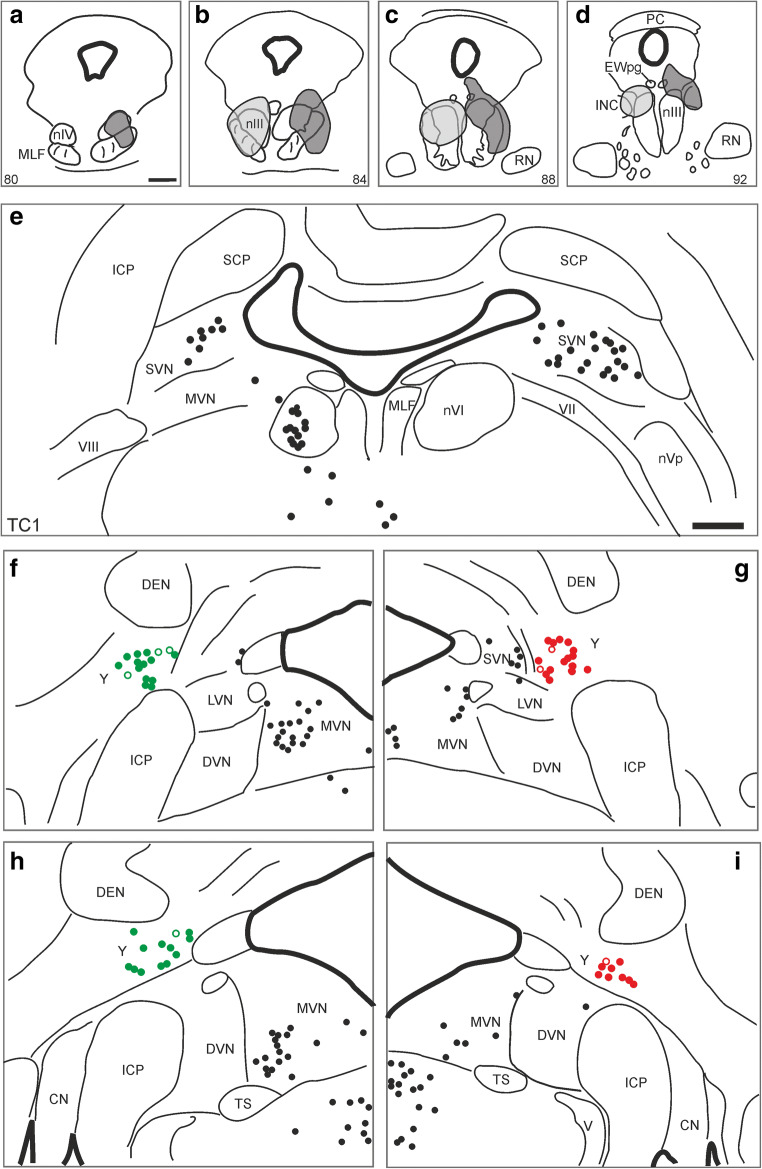
Demonstration of the tracer injection sites for two cases drawn in one series of sections through the oculomotor nucleus (nIII) region, on the left side for WGA (TC2, light grey), on the right side for CTB (TC1, dark grey) (**a**–**d**). A series of frontal sections through the medulla demonstrates the analysis of the retrogradely labelled neurons in the Y-group (Y) of the case with the unilateral CTB-injection into the right nIII. The green closed circles indicate calretinin (CR)-positive tracer–labelled neurons, the open circles CR-negative ones in Y. The red closed circles represent glutamate decarboxylase (GAD)—positive neurons, the open circles could not be judged (**f**–**i**). The black dots in all sections of **e-i** indicate tracer-labelled neurons not analysed for CR or GAD-immunoreactivity. CN, cochlear nucleus; DEN, dentate nucleus; DVN, descending vestibular nucleus; EWpg, preganglionic Edinger-Westphal nucleus; ICP, inferior cerebellar peduncle; INC, interstitial nucleus of Cajal; LVN, lateral vestibular nucleus; MVN, medial vestibular nucleus; MLF, medial longitudinal fascicle; nIV, trochlear nucleus; nVI, abducens nucleus; nVp, principal trigeminal nucleus; PC, posterior commissure; RN, red nucleus; SVN, superior vestibular nucleus; SCP, superior cerebellar peduncle; TS, nucleus of the solitary tract; V, trigeminal nerve; VII, facial nerve; VIII, vestibular nerve; Scale bar = 1 mm in **a** (applies to **a**–**d**), 1 mm in **e** (applies to **e**–**i**)

The population and location of premotor neurons in Yd is illustrated in Fig. 1b resulting from a large bilateral WGA-HRP injection covering the oculomotor (nIII) and trochlear nuclei (nIV) (case M5). Two tracer cases with smaller injections were chosen for the present study (Fig. 2). Whereas the WGA-injection of case TC2 showed some spread to the contralateral side (Fig. 2c, light grey), the CTB-injection in case TC1 was confined to one side involving nIV and lateral and dorsal portions of nIII (Fig. 2a–d, dark grey). The halo of the injection site also covered parts of the medial longitudinal fascicle (MLF), and for both cases involvement of the caudal interstitial nucleus of Cajal (INC) cannot be ruled out. In both cases, tracer injections resulted in retrograde labelling of a large population of medium-sized neurons in the Yd of both sides with a slight contralateral predominance as described in previous studies [24, 37]. In addition, numerous retrogradely labelled neurons were present in the magnocellular part of the medial vestibular nucleus (MVN) mainly contralateral and the superior vestibular nucleus (SVN) on both sides representing secondary vestibulo-ocular neurons (Fig. 2e–i) [6]. The labelling of internuclear neurons only in the contralateral abducens nucleus (nVI) confirmed the strict unilateral injection of case TC1 (Fig. 2e).

Double-immunofluorescence staining showed that all tracer-labelled neurons in Yd are ensheathed by prominent aggrecan (ACAN)–based perineuronal nets (PN) (Fig. 1c, red). In parallel sections immunoreactivity for non-phosphorylated neurofilaments (NPNF) was found in most tracer-labelled neurons (Fig. 1d, e, large arrows), but some retrogradely neurons lacked NPNF (Fig. 1d, e small arrows). In line with these observations, the analysis of neighbouring 5 μm sections containing Yd revealed that all NPNF-positive neurons are ensheathed by PN, here visualized by detection of chondroitin-sulphate proteoglycans (CSPG) (Fig. 1f, g; arrows). Strongly stained PN were also present around small neurons within the Yv, which expressed only weak NPNF-immunoreactivity, if at all (Fig. 1f,g; thin arrow in Yv).

**Fig. 3:**
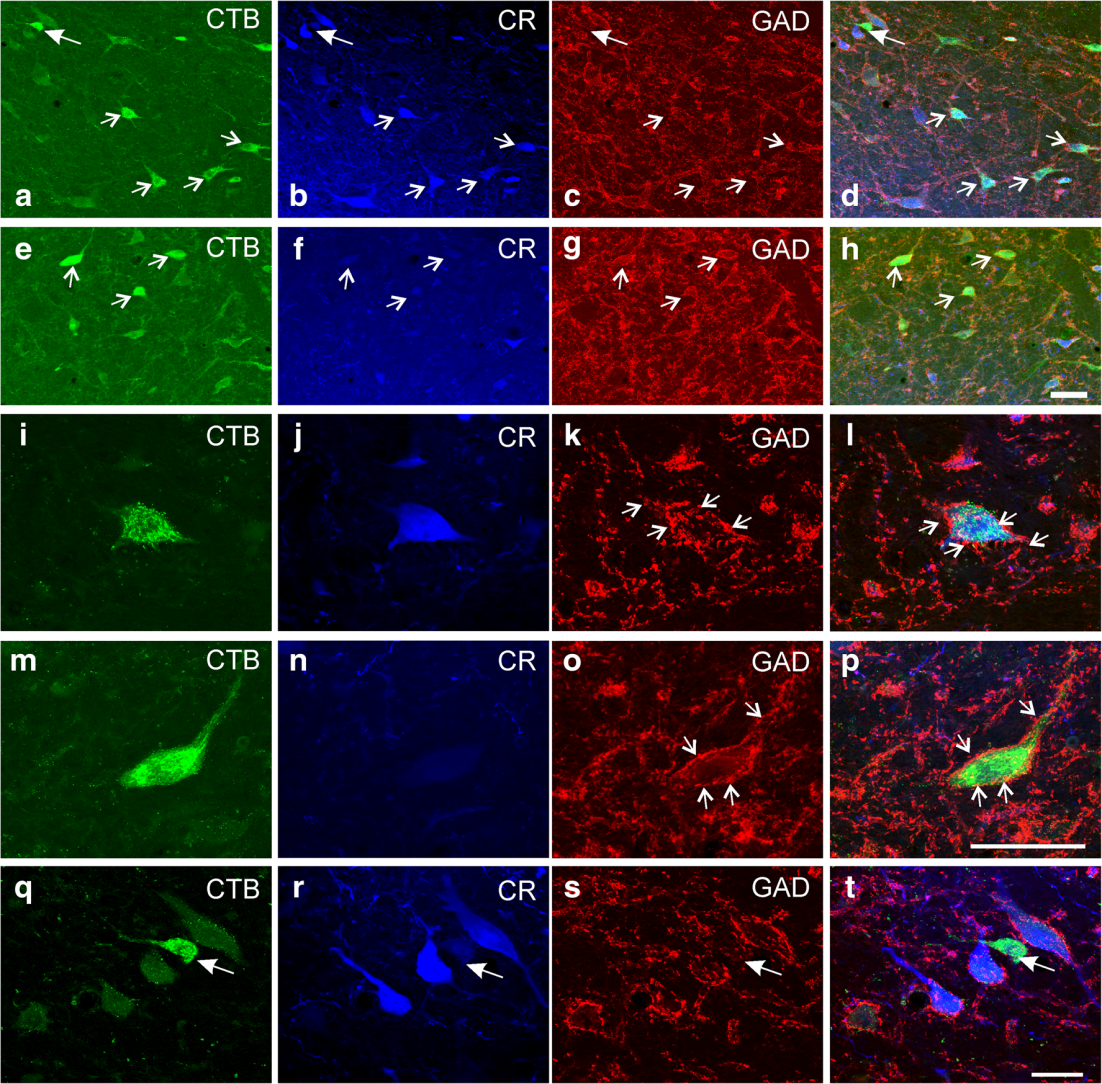
**a**–**h** Overview confocal images of double-immunofluorescence staining for cholera toxin subunit B (CTB) combined with the detection of calretinin (CR) and glutamate decarboxylase (GAD) in frontal sections through the dorsal Y-group of a monkey (TC1) who had received a unilateral tracer injection into the right oculomotor nucleus. **a**–**d** shows the staining for the contralateral side, **e**–**h** for the ipsilateral side. Note that almost all retrogradely labelled neurons (**a**, green) contain CR (**b**, blue), but not somatal GAD immunostaining (**c**, red). The arrows indicate examples of CR-positive and GAD-negative tracer-labelled neurons appearing cyan in the overlay (**d**) (**a**–**c**, small arrows). A small number of tracer-labelled neurons were found that did neither express CR nor GAD (solid arrow in **a**–**d**). On the contrary, none of tracer-labelled neurons in the ipsilateral Yd express CR (**e**, **f**, **h** arrows) but show somatal GAD-immunostaining (**g**, arrows). Detailed view of a CR-positive tracer-labelled neuron (from **a**) in the contralateral Yd, which does not express GAD in the cell body, but numerous GAD-boutons outlining the cell body (**i**–**l, **arrows). Detailed view of a CR-negative tracer-labelled neuron (from **e**) in the ipsilateral Yd with moderate GAD-immunoreactivity of the cell body also outlined by GAD-positive boutons (**m**–**p**, arrows). Detail of a tracer-labelled neuron in the contralateral Yd (from **a**) that does neither express CR nor GAD within the cell body (arrow, **q**–**t**). Scale bar = 100 μm in **h** (applies to **a**–**h**), 50 μm in **p** (applies to **i**–**p**), 50 μm in **t** (applies to **q–t**)

**Fig. 4:**
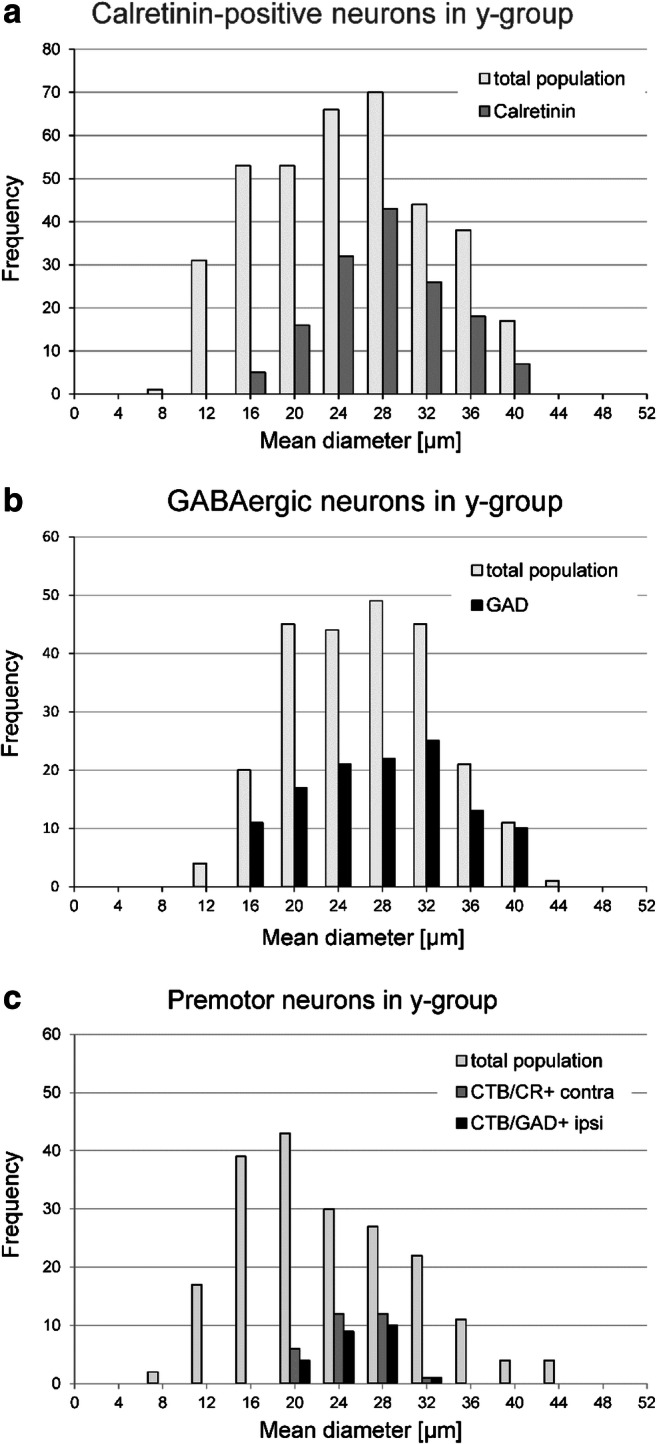
Cell size histogram of the calretinin (CR)- (**a**) and GAD-positive neurons (**b**) within the total neuron population in the dorsal Y-group (Yd) revealed from Nissl-stained sections. There is no clear indication for a bimodal population, but cell sizes range from small mean diameters of 10 μm to large cells with mean diameters of more than 40 μm. CR- and GAD-positive neurons cover almost the complete spectrum except neurons smaller than 16 μm. The lower panel shows the cell sizes of tracer-labelled CR- and GAD-positive neurons in Yd, which involve similar populations of medium-sized neurons.

The careful analysis of tracer-labelled neurons in both cases revealed that approximately 90% (91% in TC1; 87% in TC2) in the contralateral Yd contain the calcium-binding protein calretinin (CR) (Fig. 2f, h green dots; Fig. 3a, b, d thin arrows) and only few lack CR (Fig. 2f, h, open green dots; Fig. 3a, b, d solid arrow). None was found on the ipsilateral side. Tracer-labelled neurons on the ipsilateral side expressed GAD-immunoreactivity within their somata, but no CR (Fig. 2g, i, red dots; Fig. 3e–h, arrows). Open circles on the ipsilateral side in Fig. 2g, i indicate neurons whose somata could not clearly be judged. Triple-immunofluorescence staining revealed that tracer-labelled CR-positive neurons in the contralateral Yd did not express somatic GAD-immunofluorescence (Fig. 3i–l), as tracer-labelled GAD-positive neurons in the ipsilateral Yd did not express CR (Fig. 3m–p). Few tracer-labelled neurons were found, that neither contained CR nor GAD (Fig. 3q–t, arrow). Strongly GAD-positive puncta were distributed in the neuropil throughout the complete Yd, with numerous of them attached to the somata and proximal dendrites of the tracer-labelled neurons (Fig. 3i–p, arrows). The morphometric analysis revealed that CR- and GAD-positive neurons formed similar large populations (CR: 41%; GAD 49%) covering almost the whole spectrum of cell sizes in Yd except small cell bodies with less than 16 μm mean diameter (Fig. 4a, b). The analysis of tracer-labelled CR- and GAD-positive neurons revealed a more focussed population of medium-sized neurons with mean diameters between 20 and 32 μm (Fig. 4c).

**Fig. 5:**
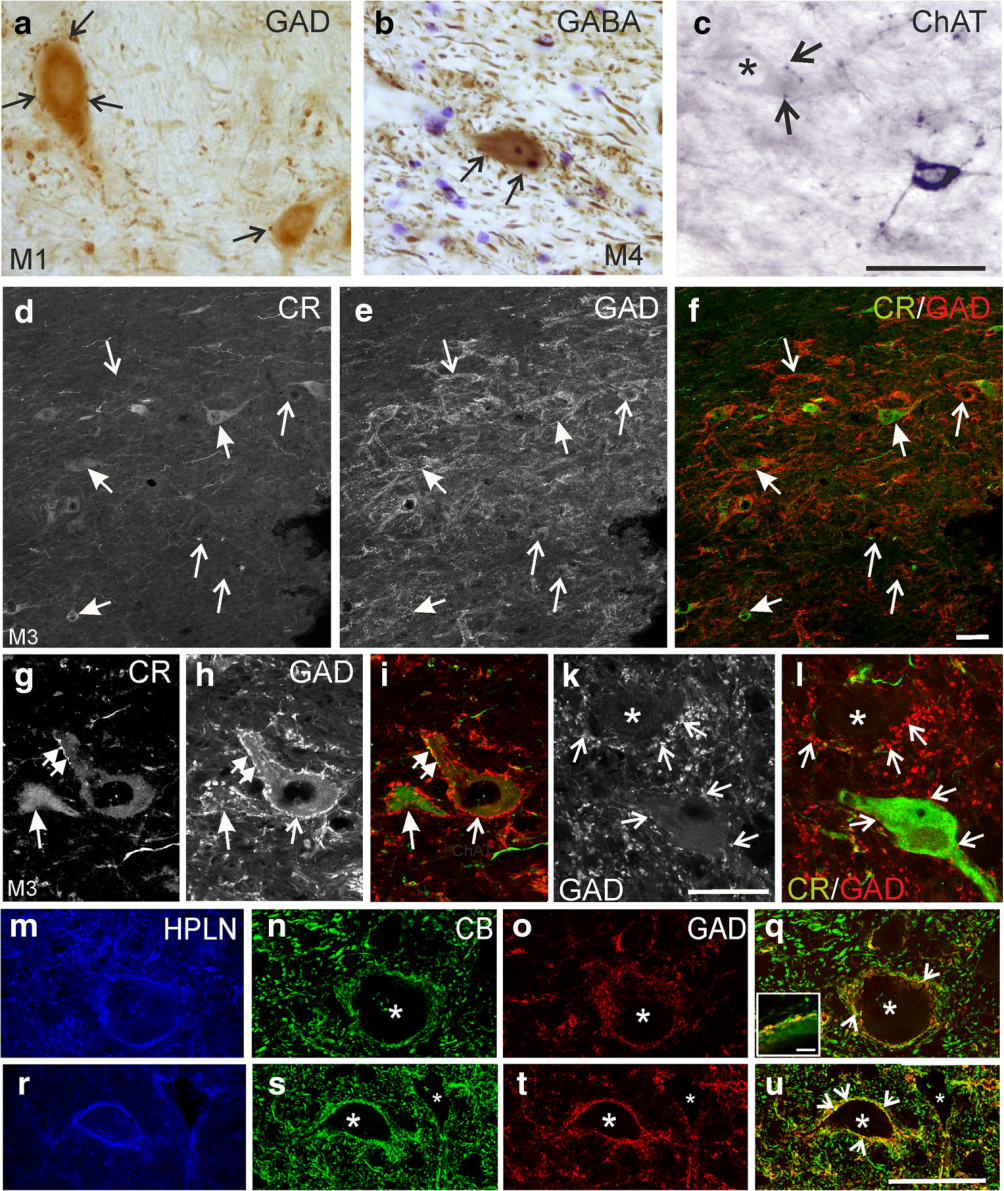
Detailed view of the dorsal Y-group (Yd) with medium-sized glutamate decarboxylase (GAD)-positive neurons (**a**, arrows) detected with a sheep antibody in thick sections. Detailed view of a medium-sized GABA-positive neuron in Yd that is contacted by GABA-positive punctate profiles (**b**, arrows). The section is counterstained with cresyl violet. Detailed view of a small ChAT-positive neuron in the Yd (**c**). Note the numerous stained axons with varicosities (arrows), some of them in close contact with ChAT-negative medium-sized neurons (asterisk). **d**–**l** Double immunostaining with rabbit CR and mouse GAD antibodies in the Yd of case M3. **d**–**f** CR-positive (solid arrows) and GAD-positive (open arrows) represent independent neuron populations without coexpression of both antigens. **g**–**h** Confocal images of detailed views reveal that CR-positive (**g**; solid arrow) as well as GAD-positive neurons (**h**; thin arrow) are associated with numerous GAD-positive boutons (**h**, **i**; small arrows). Note that all GAD-positive boutons do not coexpress CR (**g**–**i**, double arrows). **k**, **l** Some small neurons within Yd outlined by GAD-positive puncta (arrows) lack CR and GAD in their somata (**k**, **l**; asterisk). **m-q** Confocal images of putative premotor neurons in Yd ensheathed by perineuronal nets stained with antibodies against hyaluronan and proteoglycan link protein 1 (HPLN) in Yd (**m**) combined with calbindin (CB) (**n**) and GAD (**o**). **r-u** In comparison, a neuron of the dentate nucleus is shown with similar staining. Note that neurons in both nuclei (asteriks) receive a strong supply by GAD-positive boutons many coexpressing CB, appearing in yellow in the overlays (**o**, **u**, arrows) as seen in bottom left box (**q**) detail picture of membrane. These boutons most probably originate from CB-positive Purkinje cells. The coexpression is stronger in the dentate nucleus. Scale bar = 50 μm in **c** (applies to **a**–**c**); 50 μm in **f** (applies to **d**–**f**), 50 μm in **l** (applies to **g**–**l**); 50 μm in **u** (applies to **m**–**u**), 5 μm in insert of **q**

Since the GAD-immunoreactivity was only weakly expressed in cell bodies (Fig. 3o), we confirmed the presence of GABAergic neurons in the Yd by analyses of sections from previous immunoperoxidase staining with different antibodies directed against either GAD [31] or GABA [32]. Both antibodies revealed a consistent moderate GAD- or GABA-immunoreactivity in some small (less than 20 μm) and numerous medium-sized neurons in Yd (Fig. 5a, b), which were contacted by numerous immunopositive puncta most likely representing synaptic terminals (Fig. 5a, b arrows).

To validate the observation that neurons do not coexpress GAD and CR from triple-immunofluorescence, where compromises were made in choice of primary antibodies in combination with the secondary antibodies (with blue giving the poorest signal), dual immunofluorescence was performed using rabbit GAD and mouse CR antibodies only. These were detected with green and red fluorescing secondary antibodies (Fig. 5d–l). Both, CR- and GAD-positive neurons are intermingled in Yd (Fig. 5d–f; thin and solid arrows, respectively) and both populations receive a similar dense supply by GAD-positive boutons (Fig. 5h–l). Few neurons did not express any of the markers (Fig. 5k, l, asterisk) and may represent a different neuron population. One further small population of cholinergic neurons was found with ChAT-immunostaining (Fig. 5c). These small ChAT-positive neurons had mean diameters of 15 μm, clearly different from the larger-sized premotor neurons (see Fig. 4). In addition, numerous ChAT-positive varicosities were found in close contact to medium-sized neurons in Yd (Fig. 5c, asterisk, arrows). A comparative triple-immunofluorescence staining revealed a strong coexpression of calbindin (CB) in GAD-positive boutons around neurons in the dentate nucleus (Fig. 5r–u, arrows), which was less pronounced in GAD-positive terminals attached to putative premotor neurons in Yd identified by PNs (revealed with HPLN antibodies) (Fig. 5m–q, arrows).

### Expression of Potassium Channels

**Fig. 6:**
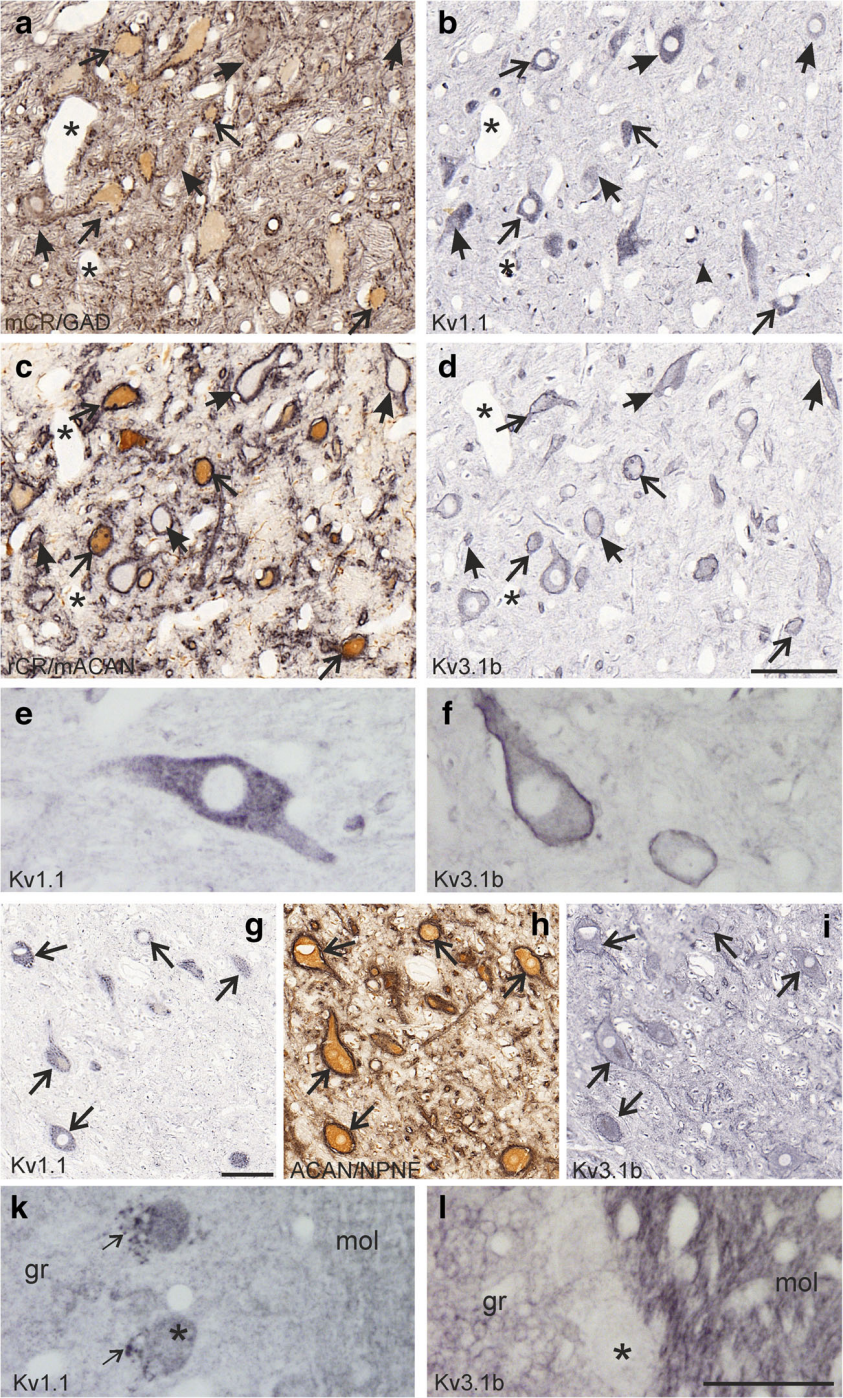
**a**–**d** Detailed views of four neighbouring paraffin sections (5 μm) through the Y-group (Yd) stained with immunoperoxidase methods for the simultaneous detection of calretinin (CR) in brown and glutamate decarboxylase (GAD) in black (**a**) or CR (brown) and aggrecan (ACAN, black) (**c**), or for the potassium channels Kv1.1 (**b**) or Kv3.1b (**d**) of case PF3. All CR- (**a**, **c**, thin arrows) and GAD-positive (**a**, solid arrows) neurons are ensheathed by ACAN-based perineuronal nets (**c**, black, thin and solid arrows), and they expressed moderate to strong Kv1.1- (**b**, arrows) and Kv3.1b-immunoreactivity (**d**, arrows). In **a**–**d** thin arrows indicate CR-positive neurons, short solid arrows putative GABAergic neurons. **e** Detail of a Kv1.1- positive neurons within Yd showing the more granular staining of the soma and proximal dendrites. **f** Detail of a Kv3.1b-positive neurons demonstrating the faint soma labelling but strong labelling of the outer cell membrane. **g**–**i** Detailed views of neighbouring paraffin sections of the magnocellular medial vestibular nucleus with putative secondary vestibulo-ocular neurons identified by their expression of nonphosphorylated neurofilament (NPNF) and perineuronal nets (ACAN) in black (**h**) (case PF2). Note that all putative vestibulo-ocular neurons express Kv1.1 and Kv3.1b (**g-i**, arrows) as the premotor neurons in Yd **(b-d**, arrows). **k** Detailed view of the cerebellar cortex stained for Kv1.1 and Kv3.1b. Note the strong Kv1.1. expression in the pinceau (arrow) at Kv1.1-positive Purkinje cells (asterisk). **l** Detail of cerebellar cortex stained for Kv3.1b. Weak Kv3.1b-immunoreactivity is present in Purkinje cells (asterisk), but strong labelling is found in the molecular (mol) and granular layer (gr). Scale bar = 100 μm in **d** applies to **a**–**d**; 50 μm in **l** (applies to **e**, **f**, **k**, **l**); 100 μm in **g** (applies to **g**–**i**)

In order to investigate the different neuronal populations in Yd for the expression of the potassium channels Kv1.1 and Kv3.1b, neighbouring thin 5 μm sections stained for either CR and ACAN, GAD and CR or Kv1.1 or Kv3.1b were analysed. Virtually, all CR-positive and GAD-positive neurons ensheathed by ACAN-based PNs expressed moderate to strong Kv1.1 immunoreactivity appearing as granular staining within the cytoplasm of the soma and proximal dendrites (Fig. 6a, b, c, arrows, e). Several neurons were visible in all neighbouring sections and allowed the additional analysis for Kv3.1b expression, which was present in all CR- and GAD-positive neurons with PNs (Fig. 6a, c, d, arrows). Unlike Kv1.1, the Kv3.1b immunoreactivity appeared as faint staining of the somata but was concentrated in the cell membrane as seen before (Fig. 6e, f) [38]. No obvious differences in the staining intensity for Kv1.1 or Kv3.1b was observed between CR-positive and GAD-positive neurons ensheathed by PN (compare Fig. 6 a with b and d). Similarly, neighbouring sections of putative secondary vestibulo-ocular neurons in the magnocellular portion of the medial vestibular nucleus (MVN) identified by PN and NPNF-expression were analysed [29]. As for Yd, all putative secondary vestibulo-ocular neurons showed immunostaining for Kv1.1 and Kv3.1b (Fig. 6g–i, arrows). The reliability of Kv immunostaining was validated in the cerebellum. GABAergic Purkinje cells show moderate Kv1.1-staining of the cell bodies, but strong staining of the pinceau, a meshwork of GABAergic terminals of basket cells associated with the axonal initial segment of Purkinje cells [39] (Fig. 6k, arrow). Only very weak Kv3.1b staining was present in the Purkinje cell, but strong immunoreactivity was expressed in the granular and molecular layers as reported before (Fig. 6l) [40]. In addition, strong Kv1.1 staining was observed in small cells, which were also distributed in the fibre tracts passing to the cerebellum, most likely representing glial cells (Fig. 6b, arrowhead).

## Discussion

The present study provides a histochemical characterization of neurons within the Yd that project to motoneurons of vertically pulling extraocular muscles with following main findings: The Yd contains two sets of projection neurons, excitatory CR-positive neurons projecting contralaterally, and an inhibitory GABAergic, but CR-negative population projecting ipsilaterally. Otherwise both populations show similar characteristics, including a strong GABAergic input, expression of nonphosphorylated neurofilaments (NPNF), wrapping with perineuronal nets, and a strong immunoreactivity for the voltage-gated potassium channels Kv1.1 and Kv3.1b. Similar histochemical profiles were found for secondary vestibulo-ocular neurons.

### Identified Cell Populations in Yd

#### CR- and GAD-Positive Premotor Neurons

A strong projection from the Yd to the oculomotor nucleus (nIII) is known from tract-tracing experiments in different species [7, 14, 23–25]. In deviation to previous findings, the present study revealed a CR-positive projection exclusively to the contralateral side [27]. The small fraction of tracer-labelled CR-positive neurons also in the ipsilateral Yd side in the previous study is attributed to tracer spread across the midline at the injection site, which did not occur in cases of the present work. A new finding was the demonstration of an ipsilateral GABAergic projection to the motonuclei. The lack of CR and GAD coexpression in the cell bodies of Yd goes along with the lack of coexpression of both proteins in synaptic terminals targeting upgaze motoneurons [26]. Accordingly, CR- and GAD-positive neurons form two independent populations in Yd, and the following assumption can be made: CR-positive neurons in Yd represent excitatory premotor neurons that may use glutamate as transmitter [8] and project through the crossing ventral tegmental tract (CVTT) to target the twitch and non-twitch motoneurons of the inferior oblique (IO) and superior rectus muscles (SR) in the contralateral nIII [14, 27, 41] and S-group [42]. This is consistent with stimulation experiments of the Y-group resulting in slow upward movements of both eyes [1] and with EPSPs in SR and IO motoneurons in contralateral nIII [43]. Similarly, recording in Yd showed an increase in firing rates during upward eye movements, with the neurons discharging in relation to upward head and eye velocity [1].

Accordingly, the GAD-positive tracer–labelled neurons must be considered as GABAergic premotor neurons, whose axons travel in the brachium conjunctivum (BC) to inhibit the motoneurons of the inferior rectus (IR) and superior oblique muscle (SO) motoneurons on the ipsilateral side during upgaze. The presence of GABA- or GAD-positive neurons in Yd was demonstrated before but was not correlated with premotor neurons [8, 44, 45]. Thereby the present study provides the anatomical basis for the findings of recording and stimulation experiments in Yd [1] and demonstrates a similar organization of crossing excitatory projections and ipsilateral inhibitory projections to respective motoneuronal groups as for the vestibulo-ocular projections of the anterior semicircular canals [8].

#### Nonpremotor Neurons in Yd

**Fig. 7:**
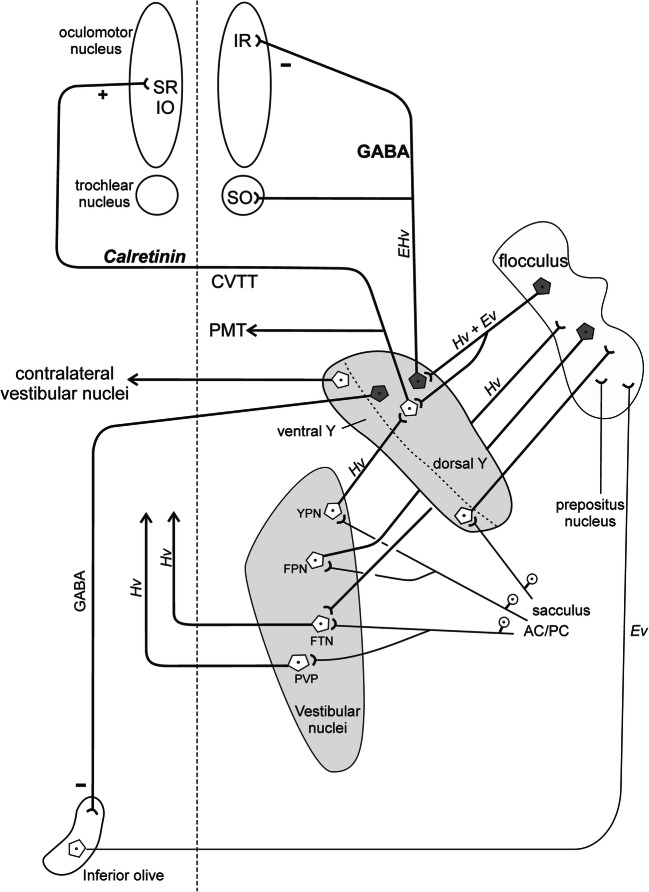
Summary diagram demonstrating the major connections of the Y-group: The dorsal Y-group (Yd) contains excitatory calretinin-positive neurons that target upgaze motoneurons in the contralateral oculomotor nucleus and GABAergic inhibitory neurons that project to downgaze motoneurons ipsilateral that may sent off projections to the paramedian tract neurons (PMT) - only drawn for the excitatory path. Both populations receive a strong GABAergic input from the flocculus. Via ‘Y-projecting interneurons’ (YPN) in the superior vestibular nucleus, the premotor neurons receive an input from the vertical semicircular canals. Floccular-projecting neurons (FPN) form another separate population that gives off collaterals to the Y-group or YPNs. In the vestibular nuclei floccular-target neurons (FTN) and position-vestibular-pause neurons (PVP) transmit head velocity signals to the motonuclei via the vestibulo-ocular reflex. Visual input is relayed to the flocculus by climbing fibres from the inferior olive and mossy fibres from the prepositus nucleus and nucleus reticularis tegmenti pontis. CVTT, crossing ventral tegmental tract, Ev eye velocity; Hv, head velocity; IR, inferior rectus muscle; IO, inferior oblique muscle; SO, superior oblique muscle; SR, superior rectus muscle

Smaller tracer-negative GAD–positive neurons in Yd may include projection neurons to the rostral dorsal cap and ventrolateral outgrowth of the contralateral inferior olive shown in rabbit with combined tracer labelling and EM analysis [46]. This projection does not arise from oculomotor projecting neurons as shown by double tracer injections in rat and rabbit, but both populations are intermingled within Yd [47] (Fig. 7).

Our finding of few primarily small ChAT-positive neurons in the Yd is consistent with other reports [48]. These cholinergic neurons differ clearly in size and morphology from those in the adjacent BIN, which coexpress GABAergic markers and send projections to the flocculus [36, 48]. It is not clear, whether ChAT-positive neurons within the Yd represent local interneurons giving rise to the abundant network of ChAT-positive nerve fibres and varicosities found in the Yd neuropil (see Fig. 5c), or whether they are projection neurons.

#### GABAergic Input to Y-Group Neurons

Anterograde tract tracing, stimulation and recording studies in monkey and cat had shown a strong projection from floccular Purkinje cells to Yd [21, 22, 49]. The covering of all tracer-labelled neurons in Yd with GAD-positive terminals, which show strong coexpression of calbindin (Fig. 5m–q, insert) expressed by Purkinje cells [50], is consistent with the expected strong GABAergic input from floccular Purkinje cells. This confirms that all premotor neurons in Yd are ‘floccular target neurons’ (FTN) consistent with electrophysiological studies in cat, which demonstrated that all identified FTNs in Yd could be antidromically activated from stimulation of the contralateral nIII [49]. But unlike FTNs in the SVN, which represent secondary vestibulo-oculomotor neurons, FTNs of the Yd are not directly targeted by primary vestibular afferents. They rather receive an indirect input via interneurons in the SVN and MVN that are activated from afferents of the anterior and posterior semicircular canals (see Fig.7) [19]. Lesions of the cerebellar loop through the flocculus may cause spontaneous upward drifts and compensating fast downward phases seen in downbeat nystagmus [3, 5]. Since 90% of floccular Purkinje cells have downward on directions, but almost none upward [21], the removal or malfunction of floccular Purkinje cells may induce an increased activity of FTNs in the Yd and SVN, which results in upward drifts of the eyes [5].

### Significance of Histochemical Properties of Yd Neurons

#### Neurofilaments

As secondary vestibulo-oculomotor neurons in the MVN and SVN the premotor neurons in Yd contain nonphosphorylated neurofilaments (NPNF) [27, 57, 29], which shape the somatic and dendritic cytoskeleton [51]. It is present in specific neuronal populations including highly active neurons of the eye movement system, e.g. omnipause neurons, saccadic burst neurons and eye muscle motoneurons [38, 52]. Due to the lack of NPNF in local interneurons, it was assumed that NPNF may contribute to long-distance projections [53]. More recent findings suggest a correlation with the amount of axonal myelination [54] rather than axonal length only.

#### Perineuronal Nets

As other functional neurons of the oculomotor system excitatory CR- and inhibitory GAD–positive neurons in Yd are wrapped by well-developed PNs also previously found in human [30, 55, 56]. PNs are lattice-like aggregates of extracellular matrix molecules with holes at synaptic contact sites [57], which ensheath distinct highly active neurons, which often contain the calcium-binding protein parvalbumin and the voltage-gated potassium channel Kv3.1b. One assumed function of PNs is a role as cation buffer [55, 58]. The expression of well-developed PNs with concomitant Kv3.1b expression in FTNs is in accordance with high spontaneous firing rates of 80–110 spikes/s recorded in squirrel monkey that are upregulated during upgaze [59]. As additional and intriguing hypothesis, PN may contribute to motor learning/adaptation of the VOR (by gain changes after wearing minimizing or magnifying googles), which is compatible with the adaptive properties of FTNs in Yd [4, 60, 61]. There is accumulating evidence that the formation of PNs does not only restrict plasticity at the end of the critical periods during postnatal development [62–64], but may also participate in the control of regained plasticity of neurons involved in adaptive processes like vestibular compensation [65]. After unilateral labyrinthectomy in adult mice, the frequency of PNs and staining intensity was strongly diminished in the lateral vestibular nuclei (mostly involved in posture), in parallel with an increase of excitatory and inhibitory synapses in the lateral vestibular nuclei of both sides. After recovery from vestibular deficits, PNs were completely restored, and in mice with genetically defective PN, vestibular compensation was accelerated [65]. The chemorepulsive protein Sema3A found as an integral component in a subset of PNs including those around neurons in the vestibular nuclei neurons may contribute to the PN mediated plasticity [66].

#### Voltage-Gated Potassium Channels Kv3.1b and Kv1.1

Alongside their role in regulating plasticity, PNs are thought to facilitate firing characteristics of fast-firing neurons via regulating expression of voltage-gated potassium channels and their localization to cell membrane [67]. Accordingly, Kv1.1 and Kv3.1b subunits form complexes with PN proteins [67]. Whereas low voltage-activated Kv1 channels regulate the resting membrane potential, threshold potential and neuronal excitability [68], high voltage activated Kv3 channels open during action potentials, actively shortening the spike duration and enabling maintained and high firing rates [69]. Together, Kv1 and Kv3 channels cooperate with Nav channels for action potential generation in highly active fast spiking neurons such as in auditory, vestibular and oculomotor systems in brainstem [38, 70]. Kv1.1 and Kv3.1b channels were similarly expressed in both, excitatory and inhibitory premotor FTNs in Yd as in secondary vestibulo-ocular neurons in the MVN. This observation suggests similar and fast firing properties for GAD-positive and CR-positive subpopulations and is in line with uniform firing patterns of Yd neurons [4]. Located between floccular Purkinje cells and oculomotor nucleus, FTNs in Yd exhibit histochemical and biophysical similarities to vestibulo-ocular neurons in vestibular nuclei—rather than to Purkinje cells (Fig. 6k, l) [38, 70].

### Proposed Circuitry

In the absence of a desired gaze shift (and Yd modulation of gaze), the eye and head movements are driven by the vestibulo-ocular reflex (VOR). A simplified model for the brainstem control of eye movements includes the direct 3-neuron VOR pathway from the vestibular afferents running in the 8th nerve, the secondary vestibular neurons in the vestibular nuclei and the motoneurons of extraocular muscles in the motonuclei (Fig. 7) [71]. Parallel indirect pathways loop through the flocculus, which include floccular-projecting neurons (FPN) in the vestibular nuclei and the ventral Y-group, both receiving direct primary afferents from vertical semicircular canals and sacculus, respectively, and projections back from the flocculus to ‘floccular target neurons’ (FTN) in the SVN and Yd. FTNs in Yd receive disynaptic excitatory signals (head velocity) from the ipsilateral vertical semicircular canals via interneurons in the anterior-lateral corner of the SVN (and MVN) [19], which are transmitted via crossing direct excitatory CR-positive and ipsilateral GABAergic projections to upgaze and downgaze motoneurons, respectively. Head-velocity signals from anterior and posterior semicircular canals reach the FTN in Yd via interneurons in the SVN (and MVN) [19]. Eye-velocity signals to Yd neurons are transmitted through the flocculus, which receives visual input via climbing fibres from the inferior olive (input from the nucleus of the optic tract) and mossy fibres (from the nucleus prepositus hypoglossi) [21, 72]. Thereby, the FTNs in the Yd transmit eye and head velocity signals to the extraocular muscles inducing upward ocular following movements. Accordingly, during pursuit Purkinje cells (mainly of the paraflocculus) provide the eye movement signal to premotor neurons, and during VOR cancellation (from flocculus) the head velocity signal via the FTNs in Yd necessary to cancel the VOR drive to motoneurons. Lesions of these circuits can result in nystagmus [3, 5].

## Conclusion

Two histochemically different populations of premotor neurons projecting to the nIII were identified in Yd: The CR-positive population represents the excitatory projection to contralateral upgaze motoneurons, whereas the GAD-positive population represents the inhibitory projection to ipsilateral downgaze motoneurons. Both populations receive a strong GABAergic input from floccular Purkinje cells indicating that all premotor neurons in Yd represent FTNs. Aside from their differing content of CR and transmitters, both premotor cell groups form a homogenous population with similar histochemical characteristics compatible with fast-firing properties, e.g. PNs and Kv channels, which were also found for secondary vestibulo-ocular neurons. The presence of well-developed PNs may also contribute to the mechanism of their adaptive capacity. The histochemical signature of premotor neurons in Yd allows the identification of the homologue cell groups in human including their inputs and will serve as basis for correlated anatomical-neuropathological studies of clinical cases with downbeat nystagmus, which is often associated with lesions of the vestibulocerebellum [3, 73]. Accordingly, the CR-positive neurons ensheathed by PN in the human Y-group most likely represent the excitatory premotor neurons targeting upgaze motoneurons in nIII [56, 74], but the pattern of GABAergic neurons and terminals as well as the ion channel expression pattern has to be studied in the future.
